# Treatment of nonocclusive mesenteric ischemia with type B aortic dissection using intra-arterial catheterization after trauma surgery: case report

**DOI:** 10.1186/s40792-017-0412-1

**Published:** 2018-01-08

**Authors:** Sho Fujiwara, Yuki Sekine, Ryuichi Nishimura, Kazuya Tadasa, Shukichi Miyazaki

**Affiliations:** 1 Department of Molecular Pathology, Tohoku University School of Medicine, Sendai, Miyagi Japan; 2 Department of Surgery, Iwate Prefectural Chubu Hospital, Kitakami, Iwate Japan; 3 Department of Vascular Surgery, Iwate Prefectural Chubu Hospital, Kitakami, Iwate Japan

**Keywords:** Nonocclusive mesenteric ischemia, Type B aortic dissection, Intra-arterial catheterization, Spasm, Trauma

## Abstract

**Background:**

Nonocclusive mesenteric ischemia (NOMI) is a mesenteric arterial spasm and intestinal ischemia. This disease is a highly lethal disease because diagnosis and decision of appropriate treatments are often difficult. Operations cannot resolve the spasms and may worsen the situation. However, the safety and effectiveness of catheterization for NOMI with aortic dissection (AD) have not yet been elucidated. Here, we report a successful case of early diagnosis and treatment of NOMI with type B AD involving the superior mesenteric artery (SMA) using the intra-arterial infusion of a vasodilator via the SMA.

**Case presentation:**

An 83-year-old man was admitted to our hospital because of abdominal pain after a motor accident. We performed intestinal resection and splenectomy for intestinal perforation and splenic hemorrhage and treated conservatively for acute AD, liver injury, renal hematoma, and pneumothorax. On postoperative day (POD) 2, the patient had localized abdominal pain. Follow-up computed tomography suggested a smaller superior mesenteric vein sign and segmental lack of enhancement in the intestinal wall and ascites without SMA occlusion. Thus, the patient was diagnosed with NOMI. Although the patient had type B AD including the SMA, we performed selective mesenteric arteriography and transcatheter papaverine infusion via the SMA and prostaglandin via the peripheral vein. Seven days post treatment, mesenteric blood flow improved and intestinal wall enhancement was restored.

**Conclusion:**

The intra-arterial infusion of a vasodilator is highly efficient and safety treatment option for NOMI with type B AD. Prompt and accurate management can prevent massive small bowel resection, and this procedure is essential in resolving a spasm independent of whether a necrotic bowel has been resected.

## Background

Nonocclusive mesenteric ischemia (NOMI) refers to a mesenteric arterial spasm and intestinal ischemia and is a highly lethal disease; the mortality rate of NOMI is reported to be 70–90% [1, 2]. Treatments include the catheter-directed and infusion of vasodilatory and antispasmodic agents and open surgery, which is often required for necrotic bowels [3]. Open surgery cannot resolve the spasms and may worsen the situation [3]. However, the safety and effectiveness of catheterization for NOMI with type B aortic dissection (AD) have not yet been elucidated [1]. Here, we report the case of a patient with NOMI having type B AD involving the superior mesenteric artery (SMA) after a motor accident who was successfully treated using the intra-arterial infusion of a vasodilator via the SMA.

## Case presentation

**Fig. 1:**
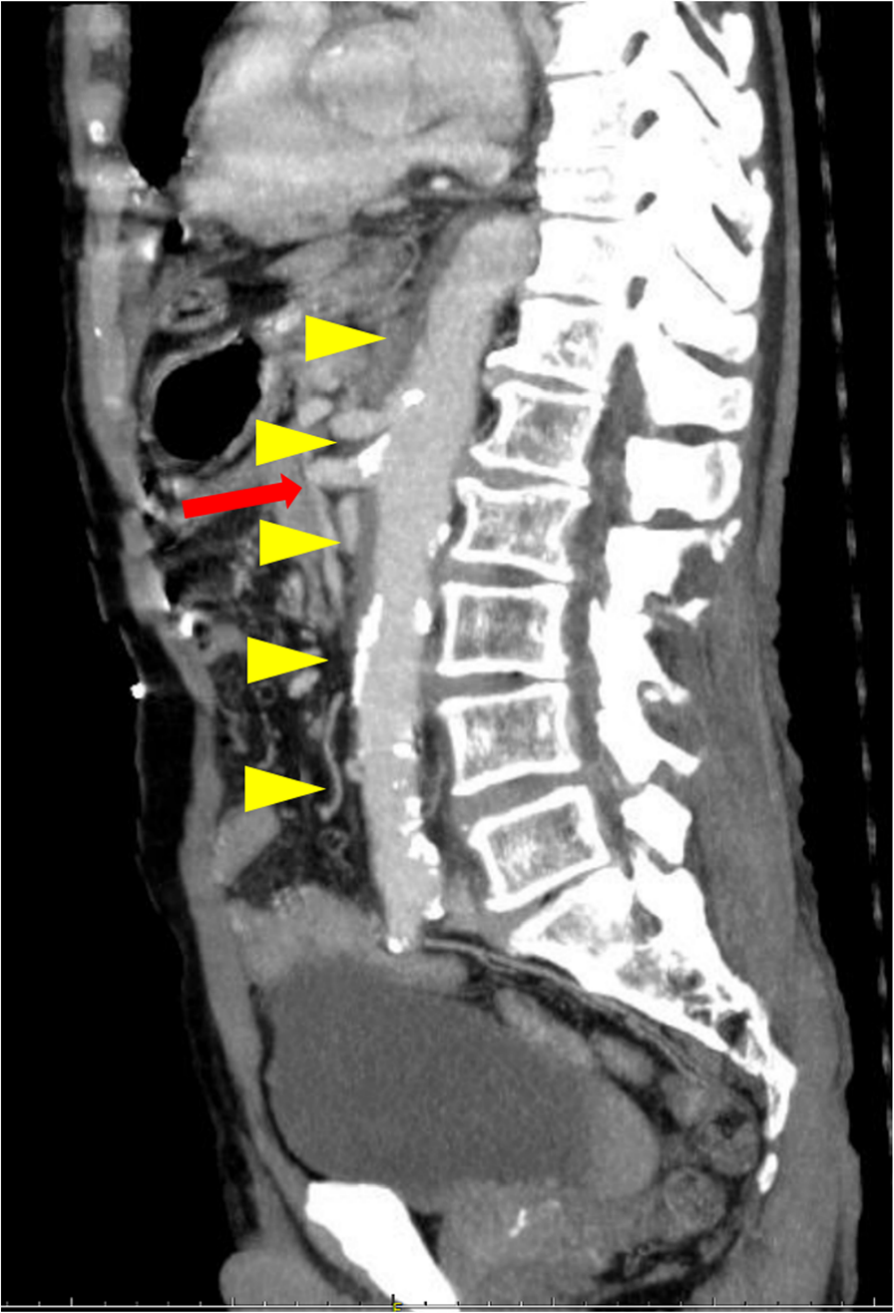
Contrast-enhanced CT indicated a type B AD (yellow arrowheads) including the SMA (red arrow)

**Fig. 2:**
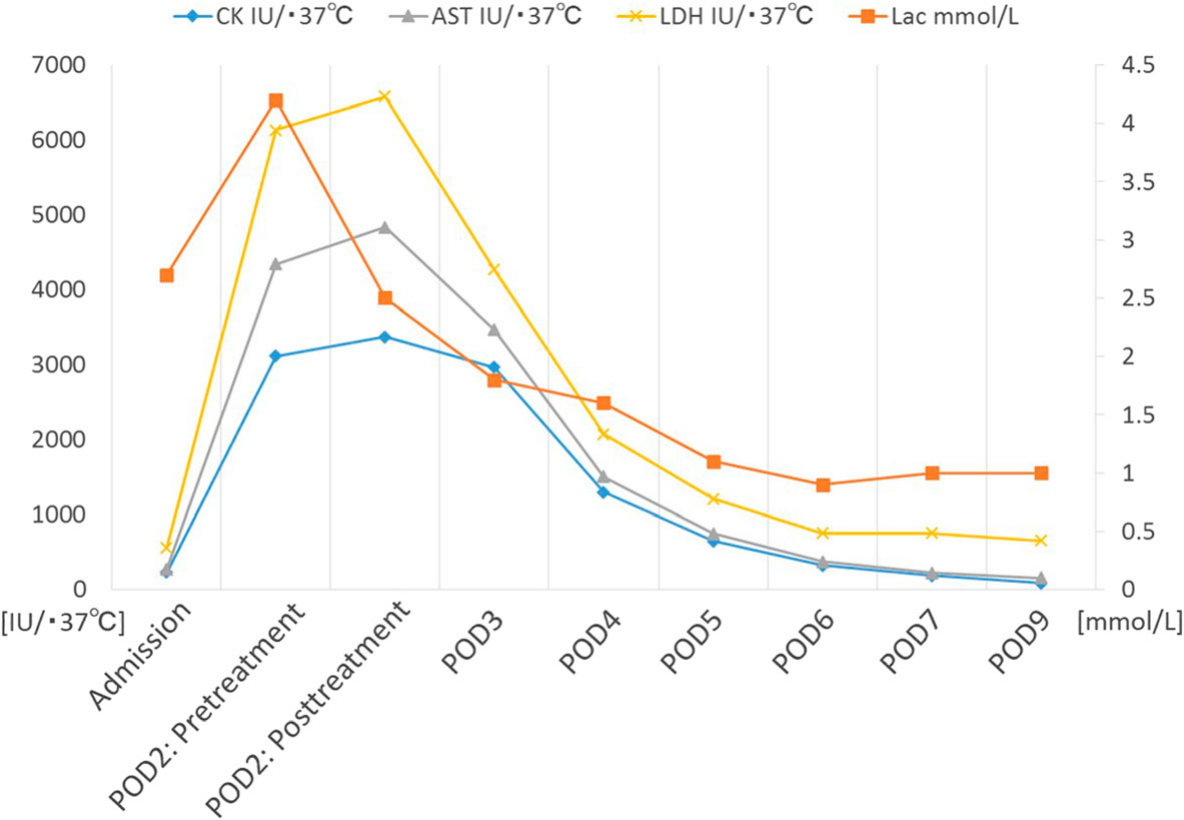
Serum creatinine kinase (CK), lactate dehydrogenase (LDH), aspartate transaminase (AST), and lactate levels were elevated on POD 2. The intra-arterial infusion of a vasodilator improved laboratory data

**Fig. 3:**
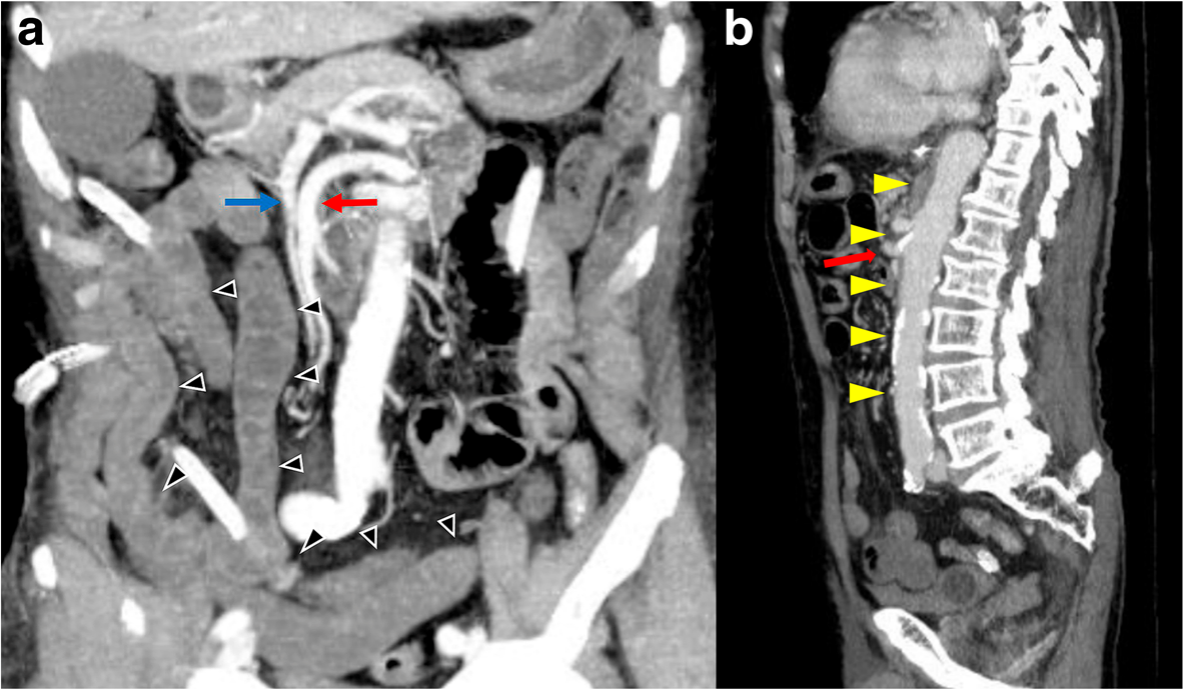
**a**, **b** Follow-up CT indicated an AD (yellow arrowheads), smaller SMV sign, and segmental lack of enhancement in the intestinal wall (arrowheads). SMV (blue arrow) was smaller than SMA (red arrow)

**Fig. 4:**
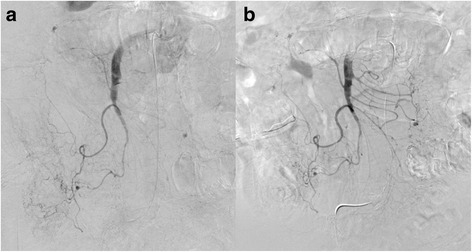
**a**, **b**: Selective mesenteric arteriography (**a**). Infusion of papaverine restored the blood flow (**b**)

**Fig. 5:**
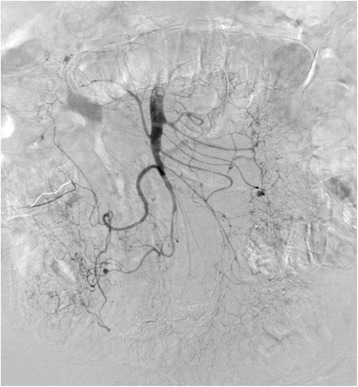
Seven days posttreatment with transcatheter papaverine infusion from the SMA and prostaglandin from peripheral vein. Mesenteric blood flow was improved

An 83-year-old man was admitted to our hospital because of abdominal pain after a motor accident. Focused assessment with sonography suggested the presence of blood in the abdomen. Contrast-enhanced computed tomography (CT) was performed (Fig. 1), which indicated intestinal perforation, acute AD, liver injury, renal hematoma, and pneumothorax. Intestinal resection (the 10 cm of ileum was resected about 50 cm from the ileoceacal valve) and splenectomy were performed, and other injuries were conservatively treated. On postoperative day 2, the patient experienced localized abdominal pain with defense and serum creatinine kinase (CK), lactate dehydrogenase (LDH), aspartate transaminase (AST), and lactate levels were elevated (Fig. 2). Then, D-dimer was 6.9 μg. Follow-up CT within an hour after the onset of symptoms revealed a smaller superior mesenteric vein sign and segmental lack of enhancement in the intestinal wall and ascites without SMA occlusion; the CT values of ischemic intestinal wall were approximately 5 to 25 Hounsfield unit (HU); ileum was 5 to 25 HU and jejunum was 65 to 90 HU (Fig. 3a). Therefore, the patient was diagnosed with NOMI, localized peritonitis, and still reversible bowel ischemia. Although selective mesenteric arteriography and intra-arterial vasodilators were considered, the patient had type B AD including the SMA (Fig. 3b), and the insertion of a catheter was considered hazardous. Furthermore, he had acute respiratory distress syndrome, which made it difficult to perform laparotomy. We decided to perform selective mesenteric arteriography with the support of a vascular surgeon 5 hours after the onset of abdominal pain (Fig. 4a). The SMA was cannulated with an 150-cm long, 0.035-in. thick, angle-type Radifocus® guidewire (Terumo, Tokyo, Japan) and a 4-French Glidecath II® shepherd-hook CJ2 (Terumo, Tokyo, Japan). The infusion of papaverine 40 mg and normal saline 19 mL after arteriography restored the blood flow (Fig. 4b). We then started transcatheter papaverine infusion (28 mg/h) via the SMA from 5-French Anthron® P-U catheter 70 cm (Toray, Tokyo, Japan) and prostaglandin (30 μg/h) via the peripheral vein. Seven days post treatment, mesenteric blood flow improved (Fig. 5) and intestinal wall enhancement was restored. We did not perform any additional operation, and the patient was discharged from our hospital.

## Discussion

We present a successful case of catheter-directed infusion into the SMA for a patient with NOMI complicated with traumatic type B AD involving the SMA. Vasodilator infusion via arterial catheter has been reported as an effective treatment for NOMI and many reports suggested efficacy of this strategies [4–7]. In 1977, Boley et al. decreased mortality rate from about 70–80% to 40% performing catheter intra-arterial infusion of papaverine [8]. Other recent study revealed vasodilator administration from SMA achieved successful treatment in 64% patients of NOMI after open heart surgery with nonsurgical treatment [7]. The strategy and management of NOMI with type B AD involving the SMA have not yet been established, and research is limited [1]. Thus, there are two overwhelming difficulties: risk from the catheterization of the dissected aorta and the accurate and prompt evaluation of NOMI.

First, the catheterization of and arterial infusion for the dissected aorta are essential, and more safety procedures were required than expected. We performed catheter-directed infusion of a vasodilator into the SMA because we believe that vasodilator therapies are the most important management option for all patients with NOMI; the resection of necrotic bowels cannot resolve spasms, which can recur by additional invasion, especially in such severe conditions [3]. Although treatments with catheter and cannulation into the SMA in the dissected aorta are essential techniques, this procedure is safe and highly efficient [3]. Recent developments in endovascular techniques and instruments enable us to perform more safety procedures than in the previous era; thoracic endovascular aortic repair and stenting for isolated SMA dissection are good examples [9]. We have to be careful in order to prevent an additional tear of the dissected aorta. However, these similar treatments have already been established as safety procedures, and cannulation of the dissected SMA has been performed during other endovascular treatments.

Second, contrast-enhanced CT considering vital signs, physical examinations, and laboratory data is essential for the accurate and prompt evaluation of NOMI [10]. CT imaging is a highly specific method for the diagnosis and evaluation of necrotic bowels [10, 11]. Although there have been reports that contrast-enhanced CT is not highly sensitive, the careful follow-up of laboratory data (lactate, CK, and LDH), abdominal pain, and vital signs can alert us regarding NOMI and bowel necrosis [11–13]. Furthermore, we apply this imaging for following of NOMI. We did not perform second-look laparotomy in this case because of the lack of intramural enhancement, and laboratory data improved after intra-arterial infusion therapy. However, if initial infusion of papaverine did not restore the spasm during angiography, we should keep in our mind and rule out other mesenteric ischemic diseases. In this case, our recommendations are arterial infusion therapy with careful observation considering laparoscopic exploratory laparotomy when bowel necrosis are suspected.

## Conclusions

The intra-arterial infusion of a vasodilator is highly efficient and can be considered as a feasible treatment option for NOMI with type B AD. Prompt and accurate management can prevent massive small bowel resection. Furthermore, this procedure is essential in resolving a spasm independent of whether a necrotic bowel has been resected.

## Abbreviations

AD: Aortic dissection
AST: Aspartate transaminase
CK: Creatinine kinase
CT: Computed tomography
HU: Hounsfield unit
LDH: Lactate dehydrogenase
NOMI: Nonocclusive mesenteric ischemia
POD: Postoperative day
SMA: Superior mesenteric artery
